# Nonlinear self-calibrated phase contrast correction in pediatric and congenital cardiovascular magnetic resonance imaging

**DOI:** 10.1186/1532-429X-18-S1-P183

**Published:** 2016-01-27

**Authors:** Erin A Paul, Ana Beatriz Solana, Amee Shah, Wyman W Lai, Anjali Chelliah

**Affiliations:** 1 Division of Pediatric Cardiology, Dept of Pediatrics, New York-Presbyterian Morgan Stanley Children's Hospital of New York, New York, NY USA; 2 GE Global Research, Garching, Germany

## Background

Phase contrast (PC) MR flow measurements are affected by phase offset errors due to local eddy currents. Performing error correction using an MR phantom prolongs each CMR study and can impede scanner workflow. Recently, a novel post-hoc nonlinear self-calibrated PC correction algorithm has been developed that can be applied to PC flow data, with equivalence to phantom correction and improved accuracy over no correction demonstrated among adults with normal cardiac anatomy (Tan ET et al, ISMRM 2014). We aim to evaluate the effect of self-calibrated correction on PC flow measurements in pediatric and congenital CMR imaging and to validate it against phantom-corrected data.

## Methods

Patients with diagnostic-quality free-breathing PC sequences of the aorta and main and branch pulmonary arteries performed at a single institution's congenital CMR program from January to June 2015 were retrospectively identified. Images were acquired using GE TwinSpeed Signa HDx 1.5T v.14 scanners with commercially available coils as part of routine clinical CMR studies. Each PC was repeated using a stationary phantom. A novel post-hoc nonlinear background phase correction method was also applied to each PC sequence. Images were processed using Medis QFlow 5.6 (Leiden, Netherlands); identical contours were applied to uncorrected, self-calibrated, and phantom images to generate three sets of net flow measurements for each PC. Intraclass correlation (ICC) was used to compare phantom-corrected and self-calibrated corrected flow data. Paired T-tests were used to compare the pulmonary-to-systemic flow ratio (Qp/Qs) calculated by phantom and self-calibrated correction to uncorrected Qp/Qs in patients without intracardiac shunts or significant valvar regurgitation.

## Results

**Figure 1:**
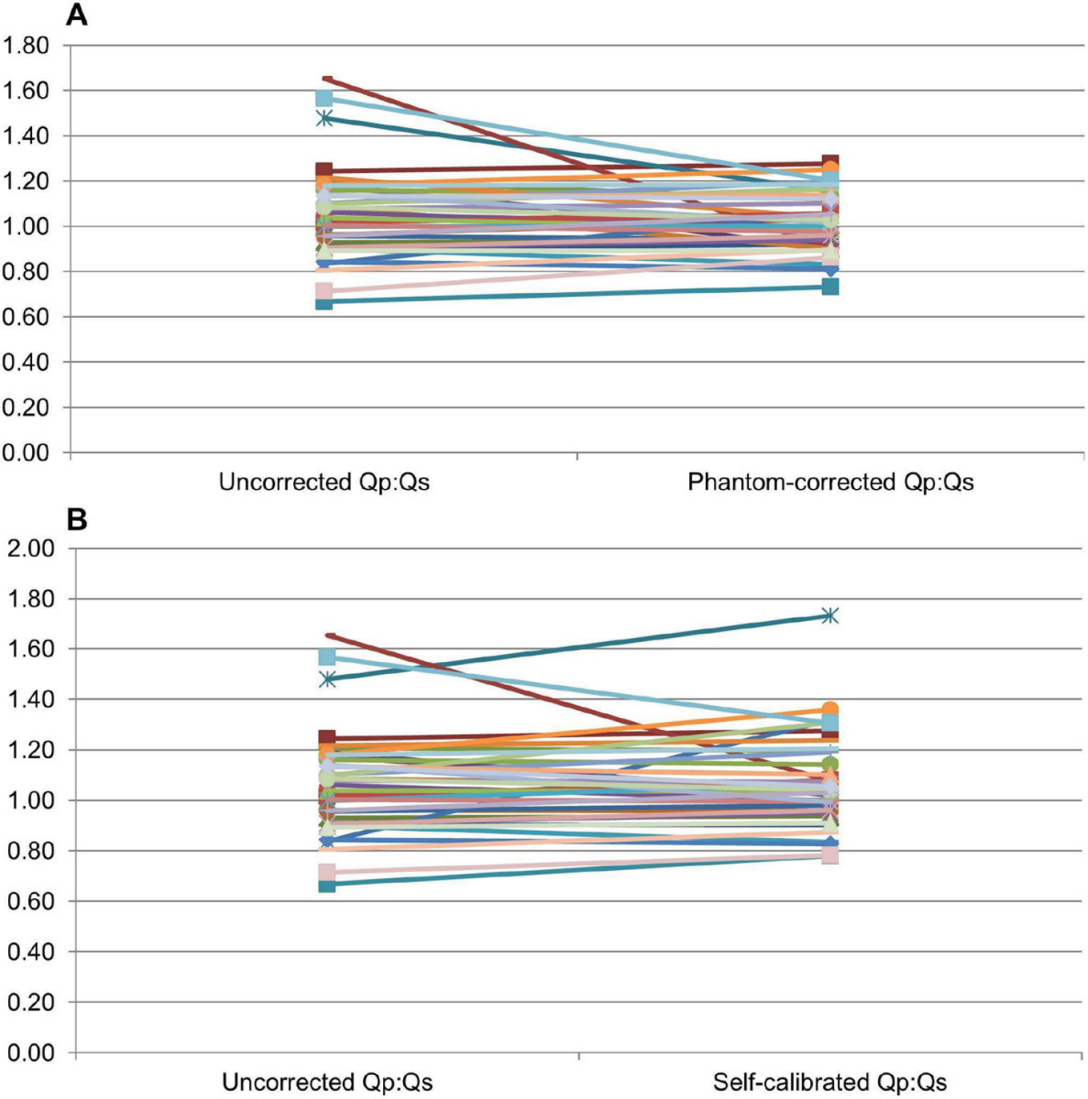
**A. Comparison of Qp:Qs calculation with and without phantom-correction**. B. Comparison of Qp:Qs calculation with and without self-calibrated correction.

227 PC sequences (100 aorta, 78 MPA, 25 RPA, and 24 LPA) from 109 patients (mean age 15 years, range 5 months-60 years) were included. The novel self-calibrated PC correction algorithm showed strong agreement with phantom-corrected data for all vessel types (ICC = 0.976), aorta (ICC = 0.950), MPA (ICC = 0.975), LPA (ICC = 0.991), and RPA (ICC = 0.992), p < 0.0001 for all ICCs. There was a nonsignificant trend towards mean Qp/Qs measurements closer to 1.0 using phantom correction compared to no correction in 39 patients without shunts or regurgitation; mean Qp/Qs was 1.06 (SD 0.20) without correction and 1.02 (SD 0.13) with phantom correction, p = 0.12 (Figure 1A). There was no significant change in mean Qp/Qs using self-calibrated correction (mean Qp/Qs 1.07, SD 0.18) versus no correction, p = 0.81 (Figure 1B).

## Conclusions

Self-calibrated PC correction showed a very high level of agreement with phantom correction among pediatric and congenital CMR patients. However, Qp/Qs measurements in patients without intracardiac shunts were not significantly changed. This study upholds our current use of phantom correction and encourages further improvements in nonlinear self-calibrated PC correction algorithms in this patient population.

